# Survey on Antioxidants Used as Additives to Improve Biodiesel’s Stability to Degradation through Oxidation

**DOI:** 10.3390/molecules28237765

**Published:** 2023-11-24

**Authors:** Elena David, Janez Kopac

**Affiliations:** 1 National Institute for Research and Development for Cryogenic and Isotopic Technologies, Street Uzinei No. 4, P.O. Râureni, P.O. Box 7, 240050 Ramnicu Valcea, Romania; 2 Faculty of Mechanical Engineering, University of Ljubljana, Askerceva 6, SI-1000 Ljubljana, Slovenia; janez.kopac@fs.uni-lj.si

**Keywords:** biodiesel stability, oxidation, antioxidants, antioxidant mechanism, cold flow

## Abstract

A major problem that limits the use of biodiesel is maintaining the fuel at the specified standards for a longer period. Biodiesel oxidizes much more easily than diesel, and the final oxidation products change its physical and chemical properties and cause the formation of insoluble gums that can block fuel filters and the supply pipes. This instability of biodiesel is a major problem and has not yet been satisfactorily resolved. Recently, the use of biodiesel has increased quite a lot, but the problem related to oxidation could become a significant impediment. A promising and cost-effective approach to improving biodiesel’s stability is to add appropriate antioxidants. Antioxidants work better or less effectively in different biodiesel fuels, and there is no one-size-fits-all inhibitor for every type of biodiesel fuel. To establish a suitable antioxidant for a certain type of biodiesel, it is necessary to know the chemistry of the antioxidants and factors that influence their effectiveness against biodiesel oxidation. Most studies on the use of antioxidants to improve the oxidative stability of biodiesel have been conducted independently. This study presents an analysis of these studies and mentions factors that must be taken into account for the choice of antioxidants so that the storage stability of biodiesel fuels can be improved.

## 1. Introduction

The main reasons for the increased interest in bioenergy are economic development, energy security, and independence [1,2], as well as the concern for reducing environmental pollution [3,4,5,6]. The commercialization of biofuels has been carried out in different ways, including the establishment of standards at the national or zonal levels, the initiation of demonstration projects, as well as the elaboration of strategies [7].

Among biofuels, biodiesel is considered the best replacement for diesel fuel [8], which is probably due to its low toxicity, good lubricity, negligible sulfur content, lower exhaust emissions, higher flash point, and possible derivation from renewable sources of raw material [5,6,9]. However, despite these advantages, large-scale use is still hampered by certain technical challenges, one of which is fuel quality [10]. The poor oxidation stability, reduced adaptability to low temperatures, and microbial degradation of biodiesel are factors that result in the degradation of biofuel.

Oxidation of biodiesel leads to the production of hydro-peroxides and carboxylic acids [11,12] that can form insoluble sediments that then clog filters or produce deposits on the fuel injector [13]. Many studies have shown that the oxidation reaction usually involves unsaturated fatty acids of the methyl ester in the composition and is accelerated by air, heat, and light [14,15,16]. It has been shown that the poor tolerance of biodiesel to low temperatures in winter is due to the crystallization process of saturated fatty acids in biodiesel, and this process causes clogging of fuel pipes and filters [17,18,19]. Microbial activities have also been found to lead to biodiesel deterioration [20,21,22,23,24]. For example, Beker et al. [25] showed that the microbial degradation of biodiesel produces an increase in the viscosity, acid number, and water content of biodiesel to values above the limit presented in the standard specifications.

To maintain the quality and stability of biodiesel, the use of synthetic chemical additives has proven to be quite effective. For example, additives such as 2-tert-butylbenzene-1,4-diol (TBHQ), 2-tert-butyl-4-methoxyphenol (BHA), and 2,6-di-tertbutyl-4-methylphenol (DBPC) were cleaved and have determined the reduction of the biofuel deterioration due to oxidation [19]. The sugar esters, such as S270 and S1570, silicon oil TSA 750, sodium dodecyl sulfate (SDS), or magnesium sulfate, presented good efficiency in preventing biofuel crystallization due to low temperatures [18].

Microbial activities could be inhibited by synthetic additives, thus preventing the microbial degradation of biofuel [25]. However, synthetic additives are expensive, and the development of cheap, biodegradable, non-toxic, and renewable additives with good efficacy is required. Substantial achievements were made in recent years, but there have been little or no comprehensive reviews of these crucial studies. Herein, some of the related studies are summarized, the state-of-the-art is established, and hypotheses and recommendations are made for further studies.

### Substitution of Fossil Fuels by Renewable Sources

**Figure 1:**
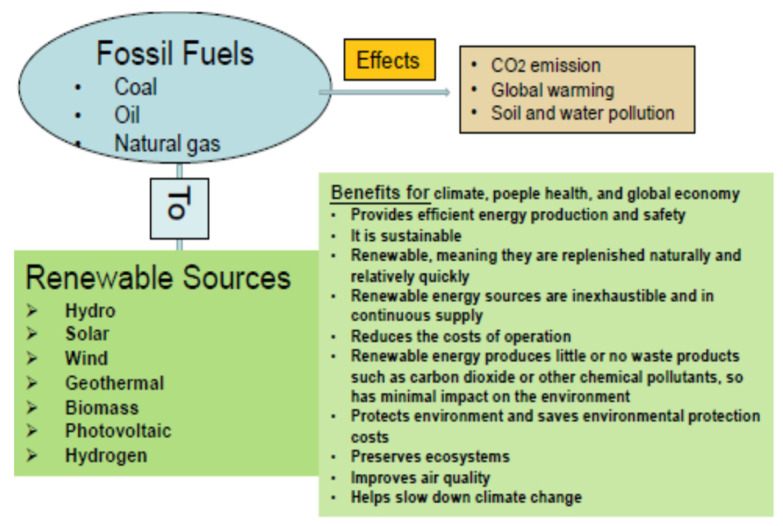
Schematic transition diagram from fossil fuels to renewable sources.

Depletion of fossil fuel sources requires finding the necessary substitute sources, considering the increase in the rate of energy consumption due to population growth, industrialization, and transportation demands. As a result, this increased demand for energy, together with the increase in greenhouse gas (GHG) emissions from the use of fossil fuels, constitutes a major challenge facing the world’s population today [26]. This issue determined the need to find sources of clean, low-cost energy, and led to the intensification of efforts based on research and revision of production paths, using new and optimized techniques, to promote clean energy production [26,27]. This will support the maintenance of energy security, limit environmental pollution, and reduce the degree of climate change [28,29]. Therefore, the current global evolution must be directed toward low-carbon sources, and most of the future energy must be provided from clean and renewable sources. Figure 1 shows a diagram of the transition from fossil fuels to renewable sources, and this transition could control the extreme climate effects generated by greenhouse gas emissions, while supporting the production and use of clean and sustainable energy.

Among the renewable sources mentioned in Figure 1, biodiesel could be considered a more sustainable and better candidate, considering its recyclability, regeneration ability, biodegradability, low sulfur content, and its low profile of greenhouse gas emissions [30]. While other renewable energies, such as wind, solar, and hydro, can mainly be used to provide electricity, energy obtained from biomass, especially biodiesel, can provide electricity and will also contribute to meeting the demand for liquid fuel from the transport sector. Since fossil fuel sources from which diesel is obtained are decreasing, new renewable sources are being sought as replacements and, thus, there is an increased demand for biodiesel use.

Although review papers have been published on the oxidative stability of biodiesel [31], methods to improve the oxidation stability of biodiesel [30], the factors affecting the oxidation process [32], the effects of antioxidants on biodiesel stability, the performance of combustion and the resulting emissions [33], a combined discussion of the impact of antioxidants on oxidative stability, and the poor cold-flow properties of biodiesel and their effects on the engine system have not been reported. Therefore, this review brings these aspects together, and the discussed parameters are presented in Table 1.

## 2. Factors Affecting the Stability to Oxidation of Biodiesel

Oxidative, hydrolytic, ketonic, and microbiological degradation are the common processes of deterioration of fatty acids. It is known that oxidation is one of primary process by which fatty acids or their esters degrade [34]. Auto-oxidation, thermal and enzymatic oxidation, or photo-oxidation are the main oxidation processes that lead to deterioration of biodiesel fuels’ quality. Among all the processes, auto-oxidation is the most common. It is a chemical process where fatty acids from biodiesel composition are degraded by oxygen in the air [35]. Usually, unsaturated fatty acids have double bonds between carbon atoms in their structure, and these double bonds can be cleaved by chemical reactions with free radicals, and the cleavage reactions also involve oxygen molecules. Typically, the oxidative degradation can cause the release of malodorous and highly volatile compounds, such as aldehydes and ketones. Since these reactions are free radical chemical reactions, they can be catalyzed by the sunlight. Factors that influence the rates of biodiesel oxidation include the chemical composition of fatty acid methyl ester (FAME) and its structure, the presence of natural antioxidants, the storage temperature and exposure to moisture, air, heat, and light, the presence of metal ions as catalysts, enzymes, and other impurities [36].

### 2.1. Influence of Feedstock Composition on Biodiesel Properties

**Figure 2:**
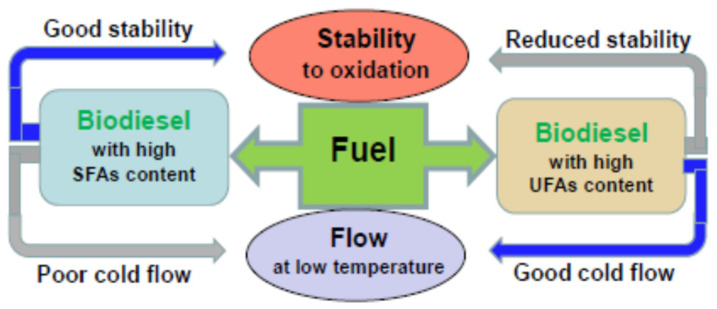
The influence of the interaction between SFAs and UFAs on the oxidation stability and cold flow of biodiesel.

Consumers, mainly those in the automotive industry, have signaled that the low oxidation stability and poor cold-flow properties of biodiesel fuels are the main causes of the degradation in biofuel quality [37,38]. Both causes are influenced by the feedstock used in the production of biodiesel, mainly by the content and composition of fatty acids in the feedstock; however, certain discrepancies have appeared between them. Biodiesel produced from raw material that contains a higher amount of saturated acids (SFAs) and a reduced amount of unsaturated fatty acids (UFAs) has a high cetane number, better oxidation stability, as well as a high calorific value, which means that the biofuel has a better quality [39]. On the other hand, this can lead to an increased viscosity at low temperatures that causes poor cold-flow properties, being in contradiction with the quality properties of the biofuel and causing fuel filter clogging and reducing the ignition efficiency in combustion engines [40]. In contrast, biodiesel containing a large amount of UFAs and a small amount of SFAs has a low cetane number, a low oxidation stability, and therefore, a worse quality. Figure 2 shows a schematic representation of how the interaction between SFAs and UFAs influences the oxidation stability and cold flow of biodiesel.

Therefore, the injection of raw biodiesel directly into combustion engines is not recommended due to these problems as well as other disadvantages, such as poor atomization and incomplete combustion, which lead to engine clogging [41]. These operability problems constitute a danger to the safety of the equipment and increase maintenance costs. These problems have been studied by researchers and several methods have been suggested to solve them, such as the use of antioxidants, mixing biodiesel with conventional diesel, pyrolysis, or micro-emulsification. Among these, the use of antioxidants to increase the oxidation stability and achieve a good cold flow has gained the attention of researchers [42,43,44].

The degradation of biodiesel through the oxidation process is proportional to the nature of the fatty acids contained in the feedstock from which the biodiesel is produced. The chemical structure of the fatty acids in the raw material is linearly correlated with the oxidative instability, even if the composition of the fatty acids differs between the types of feedstock [42]. In their study, Meira et al. [44] showed that the composition of fatty acids, moisture, glycerin content, as well as the storage conditions of the biofuel (temperature and exposure to light) are some of the factors that influence the auto-oxidation process.

The oxidation process is triggered by the loss of hydrogen atoms belonging to the allylic or bis-allylic carbon atom in the presence of an initiator, when a free radical is formed, which quickly reacts with an oxygen molecule and forms peroxyl radicals and, subsequently, propagates and forms hydro-peroxides. Hydro-peroxides formed are unstable compounds and continuously decompose, forming aldehydes and short-chain organic acids as oxidation products. These compounds progressively form insoluble gums through polymerization reactions that increase the acid value and viscosity [45,46]. Consequently, when biodiesel is oxidized, a series of changes occur that most often reduce the quality of the biofuel and, thus, the engine performance is affected.

As mentioned, fatty acids are saturated (SFAs), such as palmitic, stearic, and hydroxystearic acids, and unsaturated (UFAs), such as oleic, linoleic, palmitoleic, ricinoleic, and linolenic acids, etc. [24,28]. Although SFAs have a negligible effect compared to UFAs on the biodiesel oxidation process, the availability and cost contribute to the choice of the type of raw material used in each area for biodiesel production. Additionally, fatty acid esters and their compositions vary depending on the sources of raw materials (see Table 2).

The variation in the composition of biodiesel has direct consequences on the quality of the biofuel, the performance of the combustion engine, and the degree of exhaust emissions [32]. Feedstocks containing a high amount of SFAs showed a higher oxidative stability than those formed by more monounsaturated fatty acids (MUFAs) and polyunsaturated fatty acids (PUFAs). This could be because MUFAs and PUFAs are more prone to the oxidation process and, therefore, the inhibition effect produced by antioxidants is diminished [17].

### 2.2. Biodiesel Chemical Composition

There are two types of fatty acids, saturated (SFAs) and unsaturated (UFAs). Stearic, palmitic, and hydroxystearic acids are saturated, while oleic, linoleic, palmitoleic, linolenic, ricinoleic, and eicosenoic acids are unsaturated fatty acids [47]. The rate of oxidation of saturated fatty acids is very slow compared to the rate of oxidation of fatty acids, and their contribution to the degree of oxidation of biodiesel can be considered insignificant most of the time. Therefore, the study of the oxidation stability of the biofuel is primarily aimed at the oxidation reactions of unsaturated fatty acids. Although the value of the iodine index (IV) is an indicator that refers to the degree of unsaturation, it cannot be an indicator of oxidative stability. For example, in their study, Knothe and Razon [48] showed that the oxidation rates of biodiesel increase with the total number of bis-allylic sites (methylene CH directly adjacent to the two double bonds) in its structure, and not with the total number of double bonds or the iodine index value. The allylic position equivalent (APE) and the bis-allylic position equivalent (BAPE) values are the main parameters that show oxidation stability, and can be calculated with the relations below [49]:

APE = 2 (AC18:1 + AC18:2 + AC18:3)(1)

BAPE = (AC18:2 + AC18:3)(2)

where A represents the amount of each fatty acid compound, C18:1—oleic acid, C18:2—linoleic acid, and C18:3—alpha-linolenic acid. The BAPE value is much more representative than the APE value because a much higher oxidation rate occurs only in the bis-allylic positions [9,48]. Linoleic and linolenic acids structurally contain bis-allylic sites and are more easily subjected to oxidation than saturated fatty acids.

In other studies [15,18,50,51], the possibilities of improving the oxidation stability of biodiesel were studied by increasing the content of saturated fatty acids in the raw material using fuel filters to clog.

### 2.3. Presence of Natural Antioxidants in Biodiesel

The data of many studies show that even biodiesel containing high saturated esters loses its stability in a very short time if it contains a low amount of natural antioxidants [52,53]. Attempting to increase the oxidation stability of biodiesel, the use of plant extracts and bio-oil as antioxidant additives has been reported in a large number of studies. Fernandes et al. [54] presented the effects of ethanol extract of *Moringa oliefra* leaves as a potential antioxidant additive for biodiesel. The results obtained showed that extracts with 98% ethanol presented bigger antioxidant effects than those with 70% ethanol, having a maximum period of induction of 19.3 h. This behavior was attributed to the presence of phenolic compounds and less to the presence of the polar solvent (ethanol). Ramalingam et al. [55] analyzed the effects of *Pongamia pinnata* leaf extract on the oxidative stability of biodiesel. They showed that the induction period significantly increased when the leaf extract dosage was increased. They reported an induction period of up to 14 h, which represented a 180% increase, as compared to the biodiesel sample without a leaf extract dosage. The antioxidant activity of this additive was attributed to the presence of carotenoid and chlorophyll II of the eight components determined in the extracts. In another study, Rocha et al. [56] also dosed *Moringa* leaf extract as an additive to increase the stability of biodiesel oxidation. The increasing extract dosage determined a directly proportional increase in the induction period of the biodiesel oxidation, where a maximum induction period of 8.75 h was reported from the 4000 ppm dosage used, which represented an improvement of about 74% compared to the biodiesel sample without an extract dosage. However, the antioxidant activity of this extract was lower when compared with that of butyl hydroxyl toluene (BHT), which is a synthetic additive. The performance of the extract was also attributed to the presence of phenolic compounds, which is in agreement with some previous studies [57,58,59]. *Moringa* extract could, therefore, be used as an effective antioxidant additive for the increase in biodiesel oxidation stability. However, this plant has important economic value as a source of food and feedstock due to its nutritive and medicinal values [60,61,62,63], and this limits its possible use and commercialization as an antioxidant additive. In another study [57], the antioxidant activity of curcumin, a plant which contains β-carotene, has been investigated. The results obtained showed that curcumin extract exhibited high antioxidant activity, and an induction period of 6.35 h and 9.11 h were obtained using dosages of 500 ppm and 1500 ppm, respectively. Devi et al. [63], in their study, showed that ginger extract had good miscibility with biodiesel due to the low water content and high proportion of non-polar compounds contained in the extract. A dosage of 2000 ppm generated an induction period of 23.99 h, while a dosage of 250 ppm generated an induction period that was within the accepted standards. The antioxidant activity of this extract on the biodiesel’s stability was attributed to the presence of higher amounts of phenolic compounds in the ethanolic extract of ginger plant, as is also reported in other previous studies [64,65,66]. Spacino et al. [67] studied the effects of ethanol extracts of herbs, such as rosemary, oregano, basil, and their mixed formulations, as antioxidant additives on B100 soybean biodiesel’s stability to the oxidation process. The results showed that the oxidation process of the biodiesel with additives became endothermic and was not spontaneous, indicating that the herb extracts were effective. Of all these extracts, a mixture of 50% rosemary, 25% oregano, and 25% basil extract was the most effective. A biodiesel with a 1:1 dosage of rosemary and oregano extracts exhibited a maximum induction period of 10.18 h [67]. It was observed that the induction periods of biodiesel samples doped with rosemary, oregano, and basil extracts were higher than those biodiesel samples doped with the synthetic antioxidants, such as TBHQ, BHA, and BHT [68].

The possible use of different bio-oils from biomass were investigated regarding the antioxidant effects on biodiesel’s stability. In their study, Garcia et al. [69] extracted bio-oil from pinewood and the efficiency of bio-oil extraction and miscibility with biodiesel were investigated. They showed that the miscibility of the bio-oil with biodiesel was improved, and the induction period increased with the increasing additive dosage. A maximum induction period of 8.8 h was obtained using isopropyl acetate. The antioxidant effects are due to the presence of phenolic compounds in bio-oil composition. In their study, Gil-Lalaguna et al. [70] presented the antioxidant activity of pinewood bio-oil used as a biodiesel additive to improve its stability. The pinewood bio-oil was hydrothermally treated using different solvents, under various parameters, such as: temperature of 250, 290, and 300 °C and pressure from 4 to 11.5 MPa. The biodiesel sample with a bio-oil dosage presented improved oxidation stability by 135% when it was used as a raw bio-oil and 400% with the bio-oil dosage that was hydrothermally treated with water as a solvent at 300 °C and 8.5 MPa. Additionally, it is shown that the biodiesel sample doped with treated bio-oil remained stable for about four months due to the presence of phenolic compounds and the increase in the concentration of catechol after the hydrothermal treatment of the bio-oil. In [71], the effects of different types of bio-oil used as additives and their mixture ratios on biodiesel’s stability to oxidation were presented. The bio-oils used as additives were produced by pyrolysis of pine pellets in a semi-continuous auger reactor and pyrolysis of pine chips using a batch pyrolysis system. Various dosages were used in the biodiesel and a good miscibility of the bio-oil with biodiesel was obtained. In the temperature range of 155 to 225 °C, the oxidation stability of the biodiesel increased for doped biodiesel samples. The effects of the bio-oils obtained from malle and pine woods on biodiesel’s stability were shown by Garcia-Perez et al. [72]. The results showed that less than 20% wt. of both of the bio-oils was miscible with the biodiesel but mixing the bio-oils with 50% wt. of ethyl acetate improved the miscibility, and the induction periods for the biodiesel doped with the pine and malle bio-oils increased from 10.2 to 28.1 and 26.0 h, respectively. It can be concluded that the bio-oils obtained by biomass pyrolysis could be suitable additives to improve biodiesel’s stability to oxidation. A key factor for efficient activity of the bio-oils as biodiesel additives is the miscibility of bio-oil with biodiesel. This factor depends on the type of alcohol used as a co-solvent for bio-oil and the amount of alcohol used [73]. For example, while ethanol dissolves only about 35% wt of bio-oil, 1-butanol and 2-propanol produce higher homogenous mixtures compared to ethanol (1-butanol dissolves up to 60% wt of bio-oils, and 2-propanol dissolves up to 50%). However, not many studies focus on the economic feasibility of bio-oils used as bio-additives or on the use of suitable solvents to produce good miscibility of the bio-oils with the biodiesel, or possible modification treatments of the bio-oils to increase their miscibility without solvents. These studies must be carried out.

### 2.4. Influence of Storage Conditions on Biodiesel’s Stability

**Figure 3:**
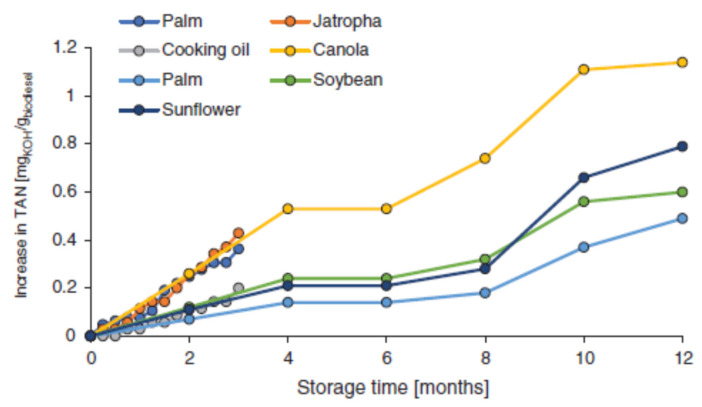
The increasing TAN value during storage for various biodiesel types [81,82].

Besides a high oxygen content, the biodiesel’s quality could be affected by other factors, such as temperature, storage time, and light exposure. The rate of oxidation reaction increases when the storage temperature is high. In their study, Xin et al. [74] showed that the oxidation reaction rate of biodiesel is much slower if the storage temperature is lowered. However, storage of biodiesel fuel at low temperatures is not used due to technical problems and, usually, biodiesel is stored at room temperature. During storage, biodiesel is not subjected to high temperatures, but when the engine is fueled, the fuel comes into contact with the spray nozzles, the piston, and the walls of the combustion chamber, which are at high temperatures, causing quick oxidation of the biodiesel and possibly producing deposits on them and causing clogging. The rate of sludge deposition depends on the fuel type and the temperature range, and the rate of sludge formation decreases when the vapor pressure of fuel increases and the partial oxygen pressure decreases. The storage time is another parameter that influences the stability of biodiesel. Ashraful et al. [75] reported that coconut oil methyl ester was deteriorated after a storage time of 12 weeks at a constant temperature and in a humid environment. Additionally, the density, viscosity, and the total acid number (TAN) of fuel increased with gum formation and sludge deposition at the end of the experiment. Batista et al. [76] determined toxic compounds, such as 2,4-decadienal or acrolein-type unsaturated aldehydes (2-heptenal and 2-octenal), during storage of soybean biodiesel in the dark, under an air environment, for a period of 5 years. In their study, Christensen and McCormick [77] studied the stability of pure biodiesel for a storage period of 12 months and of B5 and B20 mixtures over a period of 3 years. They showed that the addition of different antioxidants was effective in restoring the biodiesel’s stability, and at least 6 months of storage is possible if antioxidants were dosed at a 6 h induction time. Many studies showed a proportional correlation between the total acid number (TAN) increasing and a long storage time, as well as high temperatures and exposure to light [78]. In the study achieved by Rajendran et al. [79] it is shown that an increase in the TAN value took place when various rapeseed FAME were stored in the dark long term (52 weeks). These results were in agreement with the data presented by Yang et al. [80], who stored other kinds of commercial biodiesel for one year, such as animal fat, soybean, and canola FAMEs in fixed conditions (tight steel tanks, volume of 10 L, dark room, at −4 °C). Figure 3 shows the increasing TAN value during storage for various biodiesel types [81,82].

After 12 months of storage, the highest TAN value corresponds to Jatropha biodiesel, while the lowest corresponds to soybean biodiesel. These data emphasize, once again, that the oxidation stability is influenced by the raw material used for biodiesel production, as described in Section 2.1.

### 2.5. Presence of Metal Contaminants

In biodiesel fuels, composition was determined by the presence of transition metals in small concentrations. Some metals, such as copper, iron, zinc, and nickel, can accelerate the auto-oxidation rate, even at low concentrations. The most common metals that were detected in biodiesel are Cu, Fe, Ni, and Cr, and these metals can cause the decomposition of the peroxides into free radicals. The metal ions in lower or higher oxidation states catalyze the reaction of hydro-peroxides’ decomposition, as shown in Equations (3) and (4):

ROOH + M^n+^ → RO* + OH^−^ + M^(n+1)+^ (3)

ROOH + M^(n+1)+^ → ROO* + H^+^ + M^n+^ (4)

and free radicals such as RO* and ROO* are produced [83].

Analyzing Equations (3) and (4), it can be seen that the metal ions are regenerated, and they produce radicals such as RO* and ROO*, which cause the reduction of the induction period (IP) [84]. The catalytic effect of metals can be canceled if deactivators such as chelating compounds are used. They form complexes with metal ions and make them unavailable to initiate the oxidation reaction.

### 2.6. The Influence of Other Factors

Exposure to light is another factor that determines biodiesel degradation. Usually, the rate of the oxidation reaction increases when the biodiesel fuel is exposed to light through infiltration, which causes the deterioration of the quality of the biodiesel. The effects of light on the oxidation stability of biodiesel are influenced by the types of fatty esters in the composition of the biofuel, as well as the types of antioxidant additives used [78]. The penetration of light into the depth of the biofuel leads to the degradation of large portions of fatty acid monoalkyl esters (FAME). Pigments such as chlorophylls and pheophytins exposed to light cause photo-oxidation, while in the dark they act as antioxidants, stopping oxidation [85]. On the other hand, biodiesel that has an increased content of mono-, di-, or tri-glycerides may absorb a larger amount of water and may be exposed to hydrolytic degradation. During this process, biodiesel is converted into alcohols and free fatty acids [86]. The hydrolytic degradation process is accelerated by a high initial content of acids, especially free fatty acids, but also by the presence of moisture and heat. The water content in biodiesel decreases the calorific value, increases the corrosion rate, and creates a favorable environment for the multiplication of microbes [86], which can favor the degradation of the biofuel. The microorganisms found in biodiesel cause fuel degradation, and their amount may vary greatly. Most of the researchers mentioned that almost all variants of microorganisms, such as bacteria, fungus, and yeast, may be found in diesel fuel and biodiesel [87]. Different species have different degradation mechanisms, thus providing different results. Microorganisms’ diversity, growth rates, and patterns can be affected by fuel constituents, such as carbon and energy sources. Microbes are also commonly found in fuel storage tanks, transport systems, and fuel supply chains. Biofilm forming is triggered by microbial growth in storage tanks and pipes, which can block filters and pipelines, as well as increase pump and injection system wear. Fuel contamination shortens the filter’s life and can result in fuel starvation, engine problems, and possible damage to the fuel injection equipment [87]. This confirms that the biodegradation of hydrocarbons is an integral part of microbial life.

## 3. Remediation Methods

Problems related to the low oxidation stability of biodiesel have attracted a lot of attention from both researchers and users. Attempts were made to solve them through physical, chemical, or genetic moderation methods. A variety of techniques to stop oxidation have been analyzed, such as vacuum technology, low-temperature storage, use of inert gas for packaging, reducing the partial pressure of oxygen in contact with biodiesel, enzyme deactivation, and the use of antioxidants. Therefore, the properties of biodiesel, such as high oxidative stability, good behavior in cold flow, and the reduction of combustion emissions, could certainly be obtained by modifying the fatty acid composition through the methods mentioned above. For example, Lanjekar and Deshmukh [37] showed that physical methods such as fractionation or winterization produce a biodiesel with low oxidation stability, which makes them non-viable methods because the biodiesel produced requires the addition of antioxidant additives. Mixing biodiesel with another biodiesel made from different raw materials affects the properties of the biofuel due to the variation of fatty acids in the composition, while mixing with diesel is quite expensive and unsustainable [88]. On the other hand, the addition of branched-chain fatty alkyl esters has negative effects on the cold-flow properties, forming crystals and sediments that determine the clogging of fuel lines and blockage of filters [89]. Chemical methods, such as hydrogenation, are not viable, primarily due to the formation of biodiesel with poor cold-flow properties, leading to the occurrence of flow restriction [90].

The modification of the structure by changing the location of the unsaturation position closer to the ester head group, reducing the number of double bonds, as well as the removal of hydroxyl groups and the conversion of cis unsaturation to the trans position are also techniques used to improve the stability to oxidation of the biodiesel [91]. Sundus et al. [92] also analyzed in detail the methods used to improve the biodiesel’s stability upon oxidation. In order to avoid contact of the water with biodiesel, they showed that the use of a method based on membranes for the purification of biodiesel can be effective. Preventing or slowing down the rate of the biodiesel oxidation process with the use of antioxidant additives is the most cost-effective method compared to other techniques. The antioxidant donates an electron or hydrogen to the free radical and, thus, the oxidation reaction is neutralized. A universal antioxidant has not been found to increase the oxidation stability of all types of biodiesel. With the diversity of available antioxidants, it is necessary to find the most suitable antioxidant additives to increase the oxidation stability of each type of biodiesel.

## 4. Antioxidants

Antioxidants are chemical compounds that slow down or stop the auto-oxidation process by delaying the production of oxidants or by interrupting the multiplication of free radicals through various reactions in the auto-oxidation chain [93]. The antioxidant efficiency of an additive added to biodiesel is influenced by several factors, such as the type and number of antioxidants present, the solubility in biodiesel, the chemical structure, the potential for redox reactions, the profile of fatty acids in FAME, the position of the hydroxyl groups, etc. [94]. The addition of antioxidant additives in biodiesel is a good method to reduce or eliminate the degradation due to the ability to delay or stop the oxidation processes [94].

Natural antioxidants are usually extracted from plants and are mainly phenolic compounds that have the ability to delay auto-oxidation by inhibiting active oxygen-containing species through various mechanisms [95]. Many phenolic, flavonoid, or carotenic substances are found naturally in plants, fruits, and vegetables, including gallic acids, curcumin, tocopherols, ascorbic acid, lycopene, vanillin, cinnamic acid, etc. [96]. Although these natural antioxidants have obvious characteristics, not enough studies have been carried out to investigate their potential for increasing the oxidation stability of biodiesel [97] (Table 3). On the other hand, the use of synthetic antioxidant additives to stop the oxidative degradation of biodiesel and other fuels has been presented on a large scale [98]. The main phenolic synthetic antioxidants used to increase the stability to oxidation of biodiesel are tertiary-butylhydroquinone (TBHQ), butylated hydroxytoluene (BHT), butylated hydroxyanisole (BHA), propyl gallate (PG), octyl gallate (OG), dodecyl gallate (DG), ethoxyquin (EQ), and pyrogallol (PY) [99] (Table 4).

To increase the stability of biodiesel, natural antioxidants are more suitable, which stabilize oxygen-containing compounds in biodiesel much more easily than synthetic antioxidants. The synthetic antioxidant additives had increased efficiency, especially for compounds distilled from petroleum, such as gasoline and blends with a low level of biodiesel. It should be emphasized that synthetic antioxidants have certain disadvantages, such as high toxicity, low thermal stability, partial solubility, and high volatility, as well as a high production cost, and some of these disadvantages can be avoided by using the natural antioxidants [95]. On the other hand, it should be mentioned that the chemical structures of antioxidant compounds largely contribute to their stability and determine the nature of their reaction mechanism [100]. Table 3 and Table 4 show the standard physico-chemical properties, such as molecular mass, solubility, and melting points, as the main factors that establish the structure–antioxidant activity relationship. Therefore, antioxidant additives with a higher molecular weight, multi-hydroxyl groups (polyphenols), and that are easily soluble in biodiesel have been shown to be effective in improving biodiesel’s stability [93].

### 4.1. Natural Antioxidants

The use of natural antioxidants as fuel additives is of great interest, primarily for health reasons, with their toxicity being much reduced. Plants that have a high content of phenolic compounds can have an antioxidant effect, and vegetable oils contain natural antioxidants, such as chlorophylls, polyphenols, tocopherols, carotenoids, tocotrienols, ascorbic acid salts, lignin, etc., which fulfill the role of protectors in the oxidation process of fatty acids. A large part of these antioxidants can be decomposed or destroyed during transesterification or refining processes [101,102]. Considering this aspect, biodiesels obtained from unrefined vegetable oils have a higher amount of natural antioxidants in their composition and, consequently, a greater stability to degradation by oxidation, but do not meet other conditions necessary to be used as fuel [103]. Phenolic compounds extracted from plants, such as tocopherols, lycopene, carotenoids, astaxanthin, canthaxanthin, zeaxanthin, caffeic acid, gallic acid, ferulic acid, vanillin, sinapic acid, p-coumaric acid, cinnamic acid, eugenol, sesamol, vanillic acid, resveratrol, etc., have antioxidant properties, and are currently widely produced and marketed. Additionally, plant extracts of rosemary, sage, thyme, cloves, oregano, allspice, cinnamon, marjoram, artichoke, eucalyptus, turmeric, etc., have been used as effective antioxidants for food products [104]. However, except for tocopherols, very few studies have been conducted on the use of natural antioxidants to stop the degradation of biodiesel fuels. It was found that tocopherols show antioxidant activity only if their concentration is approximately equal to their concentration in vegetable oil, and at a higher concentration, they could act as an oxidant agent [27,105]. Further, in many studies, it has been shown that tocopherols, compared to synthetic antioxidants, have a limited antioxidant effect on biodiesel fuels, being more effective for diesel than for biodiesel produced from vegetable oils or their esters [101,102,103]. In addition, tocopherols are compounds that oxidize easily in air, and they are only stable in an inert environment (in the absence of air). The β-carotene compound is usually found in palm oil and represents a potential antioxidant. Its activity is influenced by the partial pressure of oxygen that it encounters; for example, at a higher oxygen level, it acts as an oxidant and favors the oxidation process [104]. The compounds present in small quantities, such as citric acid and polyphenols, also contribute to increasing the stability by trapping metal ions that favor the oxidation process. The oil also contains ascorbic acid, which can act as an antioxidant with a secondary role of reducing the formation of hydro-peroxides [106]. Bassil et al. and Damasceno et al. [107,108] tested the antioxidant effect of caffeic acid (CA) in a concentration of 1000 ppm in soy methyl ester and showed that CA could have antioxidant activity even after a period of three months. In addition to their role as a hydrogen donor, free radicals generated from caffeic acid form a dimer that has antioxidant properties and determines additional protection [109]. Fernandes et al. [54] demonstrated that the ethanolic extract of *Moringa oleifera* leaves has better antioxidant properties than tertiary-butylhydroquinone (TBHQ) present in biodiesel obtained from soybeans. Moser [110] studied the antioxidant effect of myricetin, a flavonoid obtained by extracting *Moringa oleifera* seeds in soybean oil methyl ester, showing that myricetin has better antioxidant activity than α-tocopherol. Additionally, Serqueira et al. [111] investigated the activity of tetrahydro-curcuminoid (a natural antioxidant obtained from curcumin) for biodiesel fuel produced from cottonseed and residual cooking oils and concluded that the antioxidant activity of tetrahydro-curcuminoid is higher than that of butylated hydroxytoluene (BHT). Medeiros et al. [112] showed that the natural extract of rosemary in ethanol has a better antioxidant performance than, for example, the synthetic antioxidant TBHQ, with the study referring to the stability of biodiesel obtained from cotton seeds. Furthermore, Spacino et al. [113] discovered an important antioxidant activity of a mixture consisting of alcoholic extracts of rosemary and oregano in a 1:1 ratio by adding it to soy methyl ester. In their study, Deyab et al. [114] observed that ethanol extracts from rosemary leaves substantially reduced the corrosion rate of aluminum present in biodiesel. More recently, other researchers [115] have demonstrated that the extracts obtained from lignocellulosic bio-oil present significant antioxidant properties, and their addition at 4% by weight to biodiesel improves the stability to degradation by oxidation by 475%. However, currently, natural antioxidants do not show high commercial success, primarily because of their higher costs.

### 4.2. Antioxidant Mechanism

The process of oxidation causes the quality degradation of biofuel, induced by auto-oxidation, photo-oxidation, thermal, and enzymatic oxidation. However, auto-oxidation is considered the most common way through which the oxidation process occurs, mainly due to the predominant amount of unsaturated fatty acids (UFAs) present in the feedstock [116]. Therefore, in this paper, the mechanism for forming the oxidation stability of the biodiesel refers on auto-oxidation, because the other forms have smaller effects.

#### Auto-Oxidation

**Figure 4:**
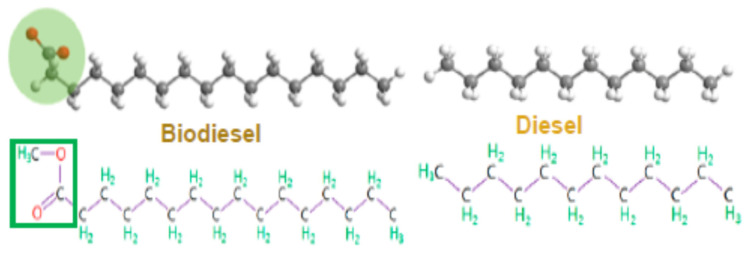
Schematic representation of the molecular structures of biodiesel and diesel.

The main difference between biodiesel and diesel is that biodiesel contains oxygen in its composition, while diesel does not. A schematic representation of the molecular structures of biodiesel and diesel is presented in Figure 4.

The presence of oxygen in the composition of biodiesel has the effect of reducing the ignition delay time. It can improve the combustion environment and ensure a more complete combustion of the fuel, which determines a reduction of the amounts of CO, PM, and other exhaust emissions. In many studies [117,118,119], it has been reported that the high oxygen content of biodiesel can substantially reduce the emissions of exhaust gases and PM from diesel engines. However, a high oxygen content enhances combustion, which results in higher combustion temperatures and increases the likelihood of combining with nitrogen, resulting in increased NOx emissions. In addition to this disadvantage, biodiesel is also subject to the degradation process through auto-oxidation, which causes its quality to deteriorate.

Auto-oxidation is an auto-catalytic reaction caused by molecular oxygen, and its speed can be increased by exposing biodiesel to air, high temperatures, or light, which leads to the formation of polymeric products that damage the quality of biodiesel. Therefore, the composition of fatty acids (saturated and unsaturated), the structural form, and the processing and storage conditions determine the intensity of auto-oxidation reactions [120]. Usually, free radicals are intermediates formed during auto-oxidation, and these are groups of atoms that carry an odd number of electrons or contain one or more unpaired valence electrons that exist freely for a fairly short time. Therefore, the free radical auto-oxidation mechanism is generally considered the fastest degradation pathway, and it involves a series of reactions that occur through initiation, propagation, and interruption of reactions, as shown below:

Initiation reaction:          RH + I → R* + IH(5)

Propagation reactions:        R* + O_2_ → ROO* (6)

ROO* + RH → ROOH + R*(7)

Interruption reactions:  ROO* + AOH → ROOH + AO^*^ (8)

R^*^ + AO* → RH + AO* (9)

ROO* + AO* → ROOA (Stable product)(10)

R* + AO* → ROA (Stable product)(11)

where I represents an initiator, air, high temperature, or light, and has the role of removing an H atom from the unsaturated fatty acid structure and generating free radicals (R*), according to Equation (5). RH represents an organic substrate, such as fatty acid monoalkyl esters (FAME), which can be oxidized. The antioxidants can slow down or inhibit the initiation reaction. The free radicals formed in the initiation reaction are very reactive and quickly combine with available O_2_, resulting in peroxy radicals (ROO*), as shown by Equation (6). The peroxy radical (ROO*) is less reactive than the R^*^ radical; however, it is reactive enough to remove another H atom from the RH structure, forming another radical, R^*^. This new R* radical can then react with molecular oxygen (O_2_), according to Equation (6), resulting a new peroxy radical (ROO*), and thus the chain reaction is propagated.

The primary or propagation chain-breaking antioxidants (AOH) can react with peroxy (ROO*) and R* free radicals, resulting in inactive products, and thus the propagation reaction does not continue—it is interrupted (according to Equations (8)–(11)). The resulting products are stable (inactive), and they do not initiate further reactions of oxidation [121].

### 4.3. Structure and Molecular Mass of the Antioxidants

**Figure 5:**
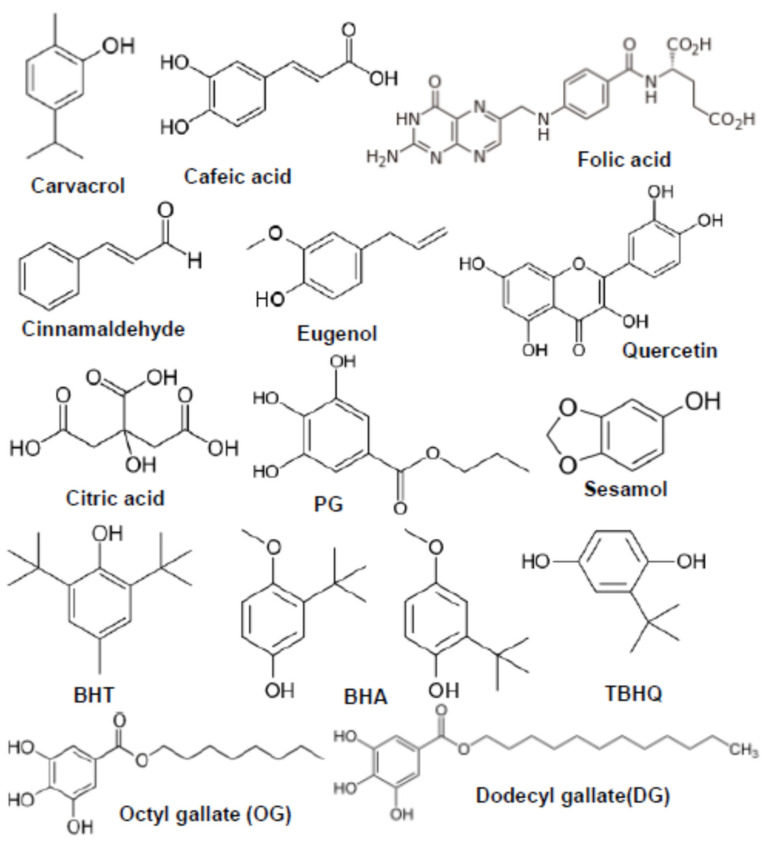
Structures of some common antioxidants.

Antioxidants act differently from one another, depending on the type and positions of the substituents attached to the aromatic ring of the compound. Figure 5 shows the molecular structure for some of the common antioxidants. Substituents such as alkyl or alkoxyl (CH_3_O) are donors of electrons, and their presence in the ortho or para positions of the aromatic nucleus of phenols is preferable in the antioxidant structure. The alkyl groups in the ortho and para positions in the structure favor the stabilization of the phenoxyl free radical generated by antioxidants and, consequently, prevent secondary reactions. Additionally, the alkyl groups in the ortho position constitute a steric hindrance and prevent the unwanted pro-oxidation reaction from taking place [122]. On the other hand, substituents such as COOH, halogens, NO_2_, and branched para-alkyl groups in the α position are acceptors of electrons, and it was found that they reduce the effectiveness of antioxidants [122]. However, most of the time, steric hindrance reduces the rate of electron release and, therefore, the antioxidant activity is diminished. The tertiary butyl groups in the structure of the BHT and BHA antioxidant molecules represent a stronger steric hindrance than that produced by the same group in the molecular structure of TBHQ, which has the effect of reducing the antioxidant activity [123]. The antioxidant activity is also influenced by the number of hydroxyl groups in the structure of the molecule. Antioxidants from the gallate series have a structure with a longer chain and possess more polyhydroxy groups (Figure 5), which determines that their antioxidant activity is higher than that of monohydroxy antioxidants, such as BHT or BHA. However, the antioxidant activity begins to decrease for the antioxidant that has more than three hydroxyl groups in its structure. In addition, the polyhydroxy substituent makes the substance partially soluble in water, which leads to a decrease in the concentration of antioxidants in the respective compound [124].

Peltzer et al. [125] showed that antioxidants that do not contain oxygen in the para position have an efficacy that increases linearly with their concentration, while antioxidants that contain para-oxygen reduce their activity with the increasing concentration. The presence of polar compounds in biodiesel, such as small amounts of methanol and ethanol, influences the performance, because the antioxidants, in the biodiesel composition or added as additives, form hydrogen bonds with the alcoholic group, slowing down the reaction of antioxidants with free radicals [126].

Antioxidants used to ensure the degradation stability for biodiesel must be able to migrate freely throughout the mass of the biofuel to reach a large number of initiation points of the auto-oxidation reaction, which are generated during storage and prevent this reaction from propagating. An oxidant with a low molecular mass can disperse well in biodiesel and can much more easily reach the sites of initiation of the auto-oxidation reaction. However, antioxidants with a low molecular mass are quite volatile, and they are lost through evaporation, which is why they are not used for long-term storage of biodiesel [127]. On the other hand, high-molecular-mass antioxidants contain more hydrogen that can be donated and have a reduced volatility, which makes them more effective for long-term biodiesel stabilization. However, antioxidants with an increased molecular mass have lower mobility, which leads to their uneven distribution in the mass of biodiesel. Thus, the concentration of these antioxidants should be ~10 times higher than the actual concentration of free radicals that initiate the auto-oxidation reaction [127]. The antioxidant performance of natural antioxidants increases, as the alkyl chain is longer (increasing the molecular mass), until a limit is reached, after which further extension of the alkyl chain length produces a rapid loss of antioxidant activity [124]. In his study, Ingendoh [128] analyzed the antioxidant performance of lower-molecular-weight BHT and higher-molecular-weight Bis-BHT by adding them to soy biodiesel, showing that Bis-BHT is more effective than BHT. Therefore, higher-molecular-weight antioxidants are preferred for long-term stabilization to oxidation of biodiesel.

### 4.4. Concentration Requirements for Antioxidants’ Use

To be effective, the antioxidants must have a minimum concentration in the biodiesel mass, from which their activity increases linearly with the increase in concentration. However, there is a final concentration value, beyond which the antioxidant activity does not improve. In most cases, for example, a higher concentration of phenolic antioxidants acts as a pro-oxidant and intensifies the degradation reaction [129,130]. The concentration interval between the minimum value (limit) and the final saturation value is known as the optimal interval, and this range is different for each type of antioxidant. Zhong and Shahidi [131] showed that the relationship between antioxidant activity and antioxidant concentrations is not linear, it follows a parabolic curve, and the minimum and maximum limits are not the same for different antioxidants—the values are higher for polar antioxidants. Chen and Luo [129] reported that the minimum critical concentration of antioxidants for a highly unsaturated biodiesel (more than 90%) is about 100 ppm to obtain a noticeable increase in the induction period (IP). The minimum limit concentration increases with the temperature, and at higher temperatures, a larger amount of antioxidant is required to obtain good biodiesel degradation stability. For example, Lapuerta et al. [132] studied the effect of temperature on the induction period of biodiesel derived from soybean oil, animal fats, and used cooking oil, using BHT as an antioxidant, and showed that at the higher temperature (130 °C), the antioxidant concentration required to reach the 8 h target was quite high (>25,000 ppm). The amount of antioxidant added in biodiesel is strongly influenced by the raw material used to produce the biodiesel and the technology. Natural antioxidants are much more sensitive to concentrations, and at higher concentrations, they show pro-oxidant effects. For example, the optimal range of the tocopherol concentration is 0.043–0.13% by weight, and at higher concentrations (>0.2%), it shows a pro-oxidant effect [34]. In addition, in [110], it was shown that the optimal concentration for α-tocopherol in soy methyl ester is in the range of 600–700 ppm and, after this range, no antioxidant activity was observed. In general, the use of higher concentrations of antioxidants in biofuels should be avoided because it could lead to an increase in the delay period in combustion and is reflected by an increase in costs.

### 4.5. Effects of Antioxidants on Biodiesel Cold-Flow Properties

Low cold-flow properties, such as cloud point (CP), pour point (PP), and cold filter plugging point (CFPP), strongly influence biodiesel’s quality parameters. These characteristics of biodiesel are much less satisfactory than those of diesel [19,133]. In low-temperature conditions, the crystallization process of biodiesel can occur, which affects the good functionality of the engine. This is due to increased viscosity, higher density, poor atomization, and vaporization, which affect the engine fuel system by clogging fuel filters and blocking fuel inlet pipes and nozzles [134]. Therefore, tracking the changes in fuel quality parameters is decisive, especially before the biofuel is under the influence of certain environmental conditions. The properties of biodiesel under low-temperature conditions must be known to estimate the longevity and better performance of the biofuel for avoiding CFPP and low-temperature filterability [90]. Thus, neglecting the properties of biofuel at low temperatures, crystals can form in its mass, and as a result, the viscosity, flow capacity, filtration, and volatility are affected, and this negatively influences the ease of starting the engine [39]. In their study, Islam et al. [135] reported that low-temperature flow is much worse for biodiesel, which contains a higher amount of saturated fatty acids (SFAs). Improving the cold flow of biodiesel can be achieved using antioxidant additives, and this is considered a conventional method that is cost-effective and sustainable. These antioxidant additives improve the flow of cold biodiesel through the process of co-crystallization of fuel crystals and thus stop the further growth of crystals [136]. In their study, Muniz et al. [137] showed that polymeric and phenolic compounds are good PP depressants and, respectively, performant antioxidant additives. Therefore, it is considered that the chemical structures of these additives consist of a series of hydrocarbons that can co-precipitate with the hydrocarbon chain of the fuel and thus prevent the development and solidification of wax crystals [138]. During the interruption of nucleation, the crystal structure the occurs, formed from a three-dimensional shape, is narrow and long-pointed, thereby preventing biodiesel filters from blockage and slowing down the growth of crystals, thus avoiding the solidification. Anoop et al. [38] observed that the CP, PP, and CFPP of biodiesel from coconut (COB) were quite high, and the biofuel was prone to the formation of crystals due to the large quantity of SFAs. By reaching an IP of 6.0 h, according to EN 21214, the biodiesel presented a better oxidative stability but showed a poor cold flow. The addition of ginger and pepper extracts increased the IP by just 3% and is steadily higher using garlic. The use of antioxidant plant extracts in different concentrations (i.e., adding 0.4% wt to 1.0% wt) reduced the CFPP by 2 °C, but a much lower decrease in the CP and PP of biodiesel was seen, as reported in [38,139]. According to the data presented, it can be emphasized that plant antioxidants have a higher efficiency in increasing the oxidation stability of biodiesel, and less so in maintaining suitable biodiesel cold-flow properties.

### 4.6. Sustainability of Using Antioxidants to Enhance Biodiesel Stability and Research Trends

The use of antioxidant additives to increase the oxidation stability and improve the cold flow of biodiesel dates back many years, and many researchers [140,141,142] have reported that the use of antioxidants is a cost-effective and sustainable way to improve the stability and performance of biodiesel. The most used antioxidants were synthetic, and among them pyrogallol (PY) proved to be the most effective and most frequently used for increasing the oxidation stability of biodiesel, followed by PG, TBHQ, BHA, and BHT, as reported in [143]. However, safety issues in the use of synthetic antioxidants, especially associated with their toxicity and high volatility, are some of the disadvantages that have led to their gradual elimination, especially in the food industry [144]. In addition, many of these antioxidants have been found to be active only at concentrations greater than 1000 ppm, and their cost is high. On the other hand, their partial solubility leads to deposits in the engine, which subsequently clogs the filters and blocks the fuel lines; thus, the maintenance costs are increased. Therefore, it can be said that the use of this class of antioxidants is not sustainable or economically viable [145] and considering the use of natural antioxidants for biodiesel stabilization would be a better option. In this context, many natural antioxidants, such as polyphenols, carotenoids, flavonoids, and amines, have been studied as additives to increase the stability of biodiesel, due to the availability of hydrogen atoms for the elimination of free radicals, high solubility, renewability, and low cost [146]. However, many of these natural antioxidants do not show good performance in terms of improving the IP of biodiesel [147]. Additionally, no important progress has been made regarding the exploration of the potential of natural antioxidants for the improvement of biodiesel cold flow. Another problem is related to the fact that the excessive use of natural edible products as antioxidant additives poses a high threat to food security by intensifying the conflict between food and fuel. In this context, few studies have presented the use of non-edible sources of antioxidants to increase the oxidation stability and poor cold-flow improvement of biodiesel. Therefore, the attempt to make this resource economically viable must be based on strategies supported by research in the field.

## 5. Conclusions

The substitution of fuels derived from oil with renewable biofuels has received special attention recently, especially through the implementation of energy policies based on energy obtained from biomass. The unsustainability of petroleum fuels is primarily due to higher toxicity and emissions with a high content of greenhouse gases. Therefore, from the reviewed literature, it appears that biodiesel as an alternative to mineral diesel is a promising option, but poor oxidation stability and poor cold flow are still major issues that limit widespread use.

Biodiesel has a reduced oxidative stability, and this creates problems, such as deposits in the engine, clogging of the fuel filter, and blockage of the supply pipes. Additionally, during long-term storage, biodiesel quality degrades due to the oxidation process. Many attempts have been made to increase the long-term storage stability of biodiesel and important progress has been seen. However, this problem is not yet completely solved, and for biodiesel-fueled engines, maintenance problems occur, which require high costs. Except for the BHT antioxidant, most of the phenolic antioxidants, both synthetic and natural, are expensive and, therefore, new high-performance, safe, ecological, and much cheaper antioxidant additives must be developed. The following conclusions emerged from this study:

- Antioxidants are chemical compounds that slow down or stop the auto-oxidation process by delaying the production of oxidants or by interrupting the multiplication of free radicals through various reactions in the auto-oxidation chain.
- The performance of antioxidants is influenced by the source from which the biodiesel fuel was obtained. Pyrogallol, for example, stood out as the most effective antioxidant for biodiesel produced from raw material with a high content of fatty acids (FFA). Biodiesel fuels that contain a small amount of natural antioxidants, such as carotenoids and tocopherols, have a reduced oxidation stability.
- The selection of antioxidants is based on their antioxidant performance, which must be high, and which is due to their good solubility, good efficiency at low concentrations, non-toxicity, and long shelf life.
- Antioxidants with a higher molecular weight ensure better stability of biodiesel over longer storage times because they contain more hydrogen atoms for donation.
- Antioxidants containing polyhydroxyl groups in their structure show higher performance compared to monohydroxyl antioxidants, such as BHT and BHA; however, no improvement in activity was observed for antioxidants with more than three hydroxyl groups in the molecular structure.
- Polar and partially fat-soluble antioxidants are more effective in maintaining the stability of biodiesel than fat-soluble antioxidants.
- The temperature, viscosity, and pH of biodiesel significantly influence the effectiveness of antioxidants. Those containing a greater number of aromatic rings and longer aliphatic chains show a higher resistance to heat. Antioxidants with high pH have a lower concentration of metal ions and improve the stability of biodiesel, and the viscosity of biodiesel greatly influences the uniform distribution of antioxidants in the mass of biodiesel.
- The use of higher concentrations of antioxidants in biofuels should be avoided, because it could lead to an increase in the delay period in combustion and reflects an increase in costs.
- The plant antioxidants have a higher efficiency in increasing the oxidation stability of biodiesel, and less so in maintaining suitable biodiesel cold-flow properties.

To increase the potential of using antioxidants as a promising method to improve biodiesel’s stability, this review suggests that other important aspects need to be studied, including: an in-depth investigation and detailed analysis of the effects of antioxidant concentrations on exhaust emissions from engines, knowledge of the evaporation rate of biodiesel mixed with antioxidants in a heated engine, the influence of antioxidants on the reduction of NOx, smoke and hydrocarbon (HC) emissions, as well as the influence of antioxidants on reduced biodiesel consumption and activation temperatures. Currently, no studies have been identified that refer to these aspects, highlighting a need for future research.

## Figures and Tables

**Table 1:** Parameters discussed.

Parameters	References
Substitution of fossil fuel by renewable sources	[26,27,28,29,30,31,32,33]
Factors affecting the stability to oxidation of biodiesel	[34,35,36]
Composition of feedstock in biodiesel production	[17,32,37,38,39,40,41,42,43,44,45,46]
Chemical composition of biodiesel	[47,48,49,50,51]
Presence of natural antioxidants in biodiesel	[52,53,54,55,56,57,58,59,60,61,62,63,64,65,66,67,68]
The effects of bio-oil on the stability to oxidation of biodiesel	[69,70,71,72,73]
Influence of storage conditions on biodiesel stability	[74,75,76,77,78,79,80,81,82,83,84]
Presence of metal contaminants	[83,84]
The influence of other factors on biodiesel stability	[78,85,86]
Remediation methods	[37,87,88,89,90,91,92]
Antioxidants	[93,94,95,96,97,98,99,100]
Natural antioxidants	[101,102,103,104,105,106,107,108,109,110,111,112,113,114,115]
Antioxidant mechanism	[116,117,118,119,120,121]
Structure and molecular mass of the antioxidants	[122,123,124,125,126,127,128]
Concentration requirement for antioxidants’ use	[34,110,129,130,131,132]
Effects of antioxidants on biodiesel cold-flow properties	[133,134,135,136,137,138,139]
Sustainability of antioxidants’ use for biodiesel stability increase and research trends	[140,141,142,143,144,145,146,147]

**Table 2:** The influence of the fatty acid composition on the biodiesel oxidation stability index (induction period, ID).

Feedstock	SFAs (%)	MUFAs (%)	PUFAs (%)	Total UFAs (%)	ID	Reference
APME	30.7	19.4	49.9	69.3	0.2	[43]
Canola	8.0	64.5	27.0	91.5	6.5	[38]
CIME	25.0	43.1	38.6	72.7	6.1	[17]
Coconut	93.8	5.0	0.82	5.82	14.2	[39]
Karanja	12.0	68.0	13.0	81.0	3.9	[41]
Jotropha	23.4	39.2	36.2	75.4	4.2	[33]
Linseed	11.4	21.8	66.2	88.0	2.2	[21]
MOME	18.6	77.1	4.3	81.4	-	[42]
Palm oil	44.6	39.0	11.0	50.0	17.0	[32]
P/Kernel	82.1	15.4	2.4	17.8	14.0	[21]
PFO	14.2	17.4	68.4	85.8	13.6	[35]
Peanut	13.0	52.0	25.0	77.0	2.0	[37]
Rapeseed	0.6	60.3	29.0	89.3	2.0	[40]
Soybean	15.0	23.1	61.9	85.0	3.0	[34]
Sunflower	10.0	21.0	62.0	83.0	1.5	[1]
SME	14.5	22.8	62.3	85.1	4.1	[17]
WCO	69.5	4.8	1.34	6.14	3.1	[36]

APME—aphanamixis polystachya methyl ester; CIME—Calophyllum inophyllum methyl ester; MOME—moringa oleifera methyl ester; MUFAs—monounsaturated fatty acid; PUFAs—poly-unsaturated fatty acid; PFO—poultry fat oil; SME—sesame oil methyl ester; WCO—waste cooking oil.

**Table 3:** Physico-chemical characteristics of some natural antioxidants (http://pubchem.ncbi.nlm.nih.gov, accessed on 25 August 2023).

Name	CID	Formula	MW	OH Groups	S (H_2_O/Alc.)	MP (°C)
					(g/mol)	
Cinnamon	637511	C_9_H_8_O	132.16	1	SS/miscible	−7.5
Sesamol	68289	C_7_H_6_O_3_	138.12	1	SS/PS	62–65
Carvacrol	10364	C_10_H_14_O	150.22	1	IS/SS	1.0
Eugenol	3314	C_10_H_12_O_2_	164.2	0	SS/SS	−7.5
Cafeic acid	689043	C_9_H_8_O_4_	180.16	4	SS/SS	225
Citric acid	311	C_6_H_8_O_7_	192.12	4	CS/SS	153
Folic acid	445858	C_10_H_10_O_4_	194.18	2	SS/SS	168–172
Curcumin	969516	C_12_H_20_O_6_	260.00	3	SS/CS	30–32
Black pepper	638024	C_17_H_19_O_3_	285.34	0	SS/SS	130
Quercetin	5280343	C_15_H_10_O_7_	302.23	5	IS/CS	316.5
Rosemary	5281792	C_18_H_16_O_8_	360.31	5	SS/SS	−20
α-tocopherol	14985	C_29_H_50_O_2_	430.7	0	IS/CS	3.0
β-carotene	573	C_40_H_56_	536.9	0	IS/SS	180
Ginger extract	6850776	C_35_H_52_O_6_	568.88	1	SS/CS	–
Grape seed extract	78577443	C_32_H_30_O_11_	590.6	9	IS/IS	–

Alc.—alcohol; MP—melting point; MW—molecular weight; S—solubility; SS—slightly soluble; CS—completely soluble; IS—insoluble.

**Table 4:** Physico-chemical characteristics of some synthetic antioxidants (http://pubchem.ncbi.nlm.nih.gov, accessed on 25 August 2023).

Name	CID	Formula	MW	OH Groups	S (H_2_O/Alc.)	MP (°C)
					(g/mol)	
PY	1057	C_6_H_3_O_3_	126.11	3	SS/CS	133
TBHQ	16043	C_10_H_14_O_2_	166.22	2	IS/CS	128
PG	4947	C_10_H_12_O_5_	212.2	3	SS/CS	130
BHA	31404	C_15_H_24_O_2_	220.35	1	IS/SS	71
OG	61253	C_15_H_22_O_5_	282.33	3	IS/SS	98–101
DG	14425	C_19_H_30_O_5_	338.44	3	IS/SS	-
BHT	24667	C_22_H_32_O_4_	360.5	1	IS/SS	70

Alc.—alcohol; MP—melting point; MW—molecular weight; S—solubility; SS—slightly soluble; CS—completely soluble; IS—insoluble; BHA—butylated hydroxyanisole; BHT—butylated hydroxytoluene; DG—dodecyl gallate; OG—octyl gallate; PG—propyl gallate; PY—pyrogallol; TBHQ—tertiary-butylhydroquinone.

## Data Availability

Data are included in the article and the relevant references.

## References

[1] Aransiola E.F., Ojumu T.V., Oyekola O.O., Madzimbamuto T.F., Ikhu-Omoregbe D.I.O. (2014). A review of current technology for biodiesel production: State of the art. Biotechnol. Bioeng..

[2] Issariyakul T., Dalai A.K. (2014). Biodiesel from vegetable oils. Renew. Sust. Energ. Rev..

[3] Demirbas A. (2008). Biofuels sources, biofuel policy, biofuel economy and global biofuel projections. Energy Convers. Manag..

[4] Singh P., Singh A. (2011). Production of liquid biofuels from renewable resources. Prog. Energy Combust. Sci..

[5] Fatt L. (2017). A review of cleaner intensification technologies in biodiesel production. J. Clean. Prod..

[6] Tiwari A., Rajesh V.M., Yadav S. (2018). Energy for sustainable development biodiesel production in micro-reactors: A review. Energy Sustain. Dev..

[7] Firoz S. (2017). A review: Advantages and disadvantages of biodiesel. Int. Res. J. Eng.Technol..

[8] Atabani A.E., Silitonga A.S., Badruddina L.A., Mahliaa T.M.I., Masjukia H.H., Mekhilef S. (2012). A comprehensive review on biodiesel as an alternative energy resource and its characteristics. Renew. Sust. Energ. Rev..

[9] De Almeida V.F., García-Moreno P.J., Guadix A., Guadix E.M. (2015). Biodiesel production from mixtures of waste fish oil, palm oil and waste frying oil: Optimization of fuel properties. Fuel Process. Technol..

[10] Anwar A., Garforth A. (2016). Challenges and opportunities of enhancing cold flow properties of biodiesel via heterogeneous catalysis. Fuel.

[11] De Carvalho A.L., Cardoso E.A., Gisele O., Teixeira L.S.G., Pepe I.M., Grosjean D.M. (2016). Carboxylic acid emissions from soybean biodiesel oxidation in the EN14112 (Rancimat) stability test. Fuel.

[12] Vieira F.S., Pasquini C. (2014). Determination of the oxidative stability of biodiesel using near infrared emission spectroscopy. Fuel.

[13] Jain S., Sharma M. (2010). Stability of biodiesel and its blends: A review. Renew. Sustain. Energy Rev..

[14] Pullen J., Saeed K. (2014). Experimental study of the factors affecting the oxidation stability of biodiesel FAME fuels. Fuel Process Technol..

[15] Kreivaitis R., Padgurskas J., Gumbytė M., Makarevičienė V., Spruogis B. (2011). The influence of oxidation on tribological properties of rapeseed oil. Transport.

[16] Serrano M., Bouaid A., Martinez M., Aracil J. (2013). Oxidation stability of biodiesel from different feedstocks: Influence of commercial additives and purification step. Fuel.

[17] Bhale P.V., Deshpande N.V., Thombre S.B. (2009). Improving the low temperature properties of biodiesel fuel. Renew. Energy.

[18] Sierra-Cantor J.F., Guerrero-Fajardo C.A. (2017). A review on the oxidation stability of biodiesel. Renew. Sustain. Energy Rev..

[19] Dwivedi G., Sharma M.P. (2014). Impact of cold flow properties of biodiesel on engine performance. Renew. Sustain. Energy Rev..

[20] Salvado J., Pasqualino J.C., Montane D. (2006). Synergic effects of biodiesel in the biodegradability of fossil-derived fuels. Biomass Bioenergy.

[21] Schleicher T., Werkmeister R., Russ W., Meyer-Pittroff R. (2009). Bioresource technology microbiological stability of biodiesel–diesel-mixtures. Bioresour. Technol..

[22] Prince R.C., Haitmanek C., Coyle C. (2008). The primary aerobic biodegradation of biodiesel B20. Chemosphere.

[23] Owsianiak M., Owsianiak M., Chrzanowski Ł., Szulc A., Staniewski J., Olszanowski A., Olejnik-Schmidt A.K., Heipieper H.J. (2009). Bioresource technology Biodegradation of diesel/biodiesel blends by a consortium of hydrocarbon degraders: Effect of the type of blend and the addition of biosurfactants. Bioresour. Technol..

[24] Makareviciene V., Janulis P. (2003). Environmental effect of rapeseed oil ethyl ester. Renew. Energy.

[25] Beker S.A., Beker S.A., da Silva Y.P., Bücker F., Cazarolli J.C., de Quadros P.D., Peralba M.D.C.R., Piatnicki C.M.S., Bento F.M. (2016). Effect of different concentrations of tert-butylhydroquinone (TBHQ) on microbial growth and chemical stability of soybean biodiesel during simulated storage. Fuel.

[26] Çamur H. (2022). Energies prediction of oxidation stability of biodiesel derived from waste and refined vegetable oils by statistical approaches. Energies.

[27] Fazal M., Jakeria M., Haseeb A., Rubaiee S. (2017). Effect of antioxidants on the stability and corrosiveness of palm biodiesel upon exposure of different metals. Energy.

[28] Nuhanovic M., Topalovic A., Culum D., Ibragic S. (2018). The effectiveness of natural and synthetic antioxidant additives on the oxidation stability of biodiesel synthesized from fresh and waste sunflower oilesized from fresh and waste sunflower oil. Orbital Electron. J. Chem..

[29] Silitonga A.S. (2017). A comparative study of biodiesel production methods for *Reutealis trisperma* biodiesel. Energy Sources Part A Recover. Util. Environ. Eff..

[30] Hazrat M.A. (2021). Techniques to improve the stability of biodiesel: A review. Environ. Chem. Lett..

[31] Monirul I.M. (2017). Influence of poly(methyl acrylate) additive on cold flow properties of coconut biodiesel blends and exhaust gas emissions. Renew. Energy.

[32] Bukkarapu K.R., Krishnasamy A. (2021). A critical review on available models to predict engine fuel properties of biodiesel. Renew. Sustain. Energy Rev..

[33] Sorate K.A., Bhale P.V. (2015). Biodiesel properties and automotive system compatibility issues. Renew. Sustain. Energy Rev..

[34] Kochhar S.P., Scott G. (1993). Deterioration of edible oils, fats and foodstuffs. Atmospheric Oxidation and Antioxidants.

[35] Shahidi F., Zhong Y. (2010). Lipid oxidation and improving the oxidative stability. Chem. Soc. Rev..

[36] Yaakob Z., Narayanan B.N., Padikkaparambil S., Suryaunni K., Akbar P. (2014). A review on the oxidation stability of biodiesel. Renew. Sustain. Energy Rev..

[37] Lanjekar R.D., Deshmukh D. (2016). A review of the effect of the composition of biodiesel on NOx emission, oxidative stability and cold flow properties. Renew. Sustain. Energy Rev..

[38] Kivevele T.T., Huan Z. (2013). Effects of antioxidants on the cetane number, viscosity, oxidation stability, and thermal properties of biodiesel produced from nonedible oils. Energy Technol..

[39] Sia C.B., Kansedo J., Tan Y.H., Lee K.T. (2019). Evaluation on biodiesel cold flow properties, oxidative stability and enhancement strategies: A review. Biocatal. Agric. Biotechnol..

[40] Adewale P., Dumont M.J., Ngadi M. (2015). Recent trends of biodiesel production from animal fat wastes and associated production techniques. Renew. Sustain. Energy Rev..

[41] Pantoja S.S., Da Conceiçao L.R.V., Da Costa C.E.F., Zamian J.R., Da Rocha Filho G.N. (2013). Oxidative stability of biodiesels produced from vegetable oils having different degrees of unsaturation. Energy Convers. Manag..

[42] Patra J.K., Kim S.H., Hwang H., Choi J.W., Baek K. (2015). Volatile compounds and antioxidant capacity of the bio-oil obtained by pyrolysis of Japanese red pine (*Pinus densif lora Siebold* and *Zucc*.). Molecules.

[43] Rashed M.M. (2015). Stability of biodiesel, its improvement and the effect of antioxidant treated blends on engine performance and emission. RSC Adv..

[44] Meira M., Santana P.M.B., Araújo A.S., Silva C.L., Filho J.R.L.L., Ferreira H.T. (2014). Oxidative degradation and corrosiveness of biodiesel. Corrosion Rev..

[45] Donoso D., Bolonio D., Lapuerta M., Canoira L. (2020). Oxidation stability: The bottleneck for the development of a fully renewable Biofuel from wine industry waste. ACS Omega.

[46] Ondul E., Dizge N., Keskinler B., Albayrak N. (2015). Biocatalytic production of biodiesel from vegetable oils. Biofuels Status Perspect..

[47] Ardhiah H.H., Ong H.C. (2017). A review on latest developments and future prospects of heterogeneous catalyst in biodiesel production from non-edible oils. Renew. Sustain. Energy Rev..

[48] Knothe G., Razon L.F. (2017). Biodiesel fuels. Prog. Energy Combust. Sci..

[49] Knothe G. (2002). Structure indices in FA chemistry. How relevant is the iodine value?. J. Am. Oil Chem. Soc..

[50] Kumar N. (2016). Oxidative stability of biodiesel: Causes, effects and prevention. Fuel.

[51] McCormick R., Ratcliff M., Moens L., Lawrence R. (2007). Several factors affecting the stability of biodiesel in standard accelerated tests. Fuel Process Technol..

[52] Lima R.P., da Luz P.T.S., Braga M., Batista P.R.d.S., da Costa C.E.F., Zamian J.R., Nascimento L.A.S.D., Filho G.N.d.R. (2017). Murumuru (*Astrocaryum murumuru Mart*.) butter and oils of buriti (*Mauritia flexuosa Mart*.) and pracaxi (*Pentaclethra macroloba* (Willd.) Kuntze) can be used for biodiesel production: Physico-chemical properties and thermal and kinetic studies. Ind. Crops Prod..

[53] Gureev A.A., Prigulskii G.B. (1985). Thermooxidative stability of diesel fuels. Chem. Technol. Fuels Oil.

[54] Fernandes D.M., Sousa R.M., de Oliveira A., Morais S.A., Richter E.M., Muñoz R.A. (2015). Moringa oleifera: A potential source for production of biodiesel and antioxidant additives. Fuel.

[55] Ramalingam S., Govindasamy M., Ezhumalai M., Kaliyaperumal A. (2016). Effect of leaf extract from Pongamia pinnata on the oxidation stability, performance and emission characteristics of calophyllum biodiesel. Fuel.

[56] Rocha Morais França F., dos Santos Freitas L., Ramos A.L.D., da Silva G.F., Brandão S.T. (2017). Storage and oxidation stability of commercial biodiesel using *Moringa oleifera Lam* as an antioxidant additive. Fuel.

[57] De Sousa L.S., De Moura C.V.R., De Oliveira J.E., De Moura E.M. (2014). Use of natural antioxidants in soybean biodiesel. Fuel.

[58] Nascimento J.A., Araújo K.L., Epaminondas P.S., Pontes A.L., Souza A.L., Queiroz N., Souza A.G. (2014). Ethanolics extracts of Moringa: Antioxidant effect in soybean oil by PDSC and Rancimat. J. Therm. Anal. Calorim..

[59] Vats S., Gupta T. (2017). Evaluation of bioactive compounds and antioxidant potential of hydroethanolic extract of *Moringa oleifera Lam*. from Rajasthan, India. Physiol. Mol. Biol. Plants.

[60] Bolarinwa I.F., Aruna T.E., Raji A.O. (2019). Nutritive value and acceptability of bread fortified with moringa seed powder. J. Saudi Soc. Agric. Sci..

[61] Omotoso G.O., Gbadamosi I.T., Olajide O.J., Dada-Habeeb S.O., Arogundade T.T., Yawson E.O. (2018). *Moringa oleifera* phytochemicals protect the brain against experimental nicotine-induced neurobehavioral disturbances and cerebellar degeneration. Pathophysiology.

[62] Gupta S., Jain R., Kachhwaha S., Kothari S.L. (2018). Nutritional and medicinal applications of Moringa oleifera Lam.-Review of current status and future possibilities. J. Herb. Med..

[63] Devi A., Das V.K., Deka D. (2017). Ginger extract as a nature based robust additive and its influence on the oxidation stability of biodiesel synthesized from non-edible oil. Fuel.

[64] Gopalakrishnan L., Doriya K., Kumar D.S. (2016). Moringa oleifera: A review on nutritive importance and its medicinal application. Food Sci. Hum. Wellness.

[65] Djikeng F.T., Womeni H.M., Anjaneyulu E., Karuna M.S.L., Prasad R.B.N., Linder M. (2018). Effects of natural antioxidants extracted from Cameroonian ginger roots on the oxidative stability of refined palm olein. Eur. Food Res. Technol..

[66] Ganji S., Sayyed-Alangi S.Z. (2017). Encapsulation of ginger ethanolic extract in nanoliposome and evaluation of its antioxidant activity on sunflower oil. Chem. Pap..

[67] Spacino K.R., Borsato D., Buosi G.M., Chendynski L.T. (2015). Determination of kinetic and thermodynamic parameters of the B100 biodiesel oxidation process in mixtures with natural antioxidants. Fuel Process Technol..

[68] Buosi G.M., Da Silva E.T., Spacino K., Silva L.R.C., Ferreira B.A.D., Borsato D. (2016). Oxidative stability of biodiesel from soybean oil: Comparison between synthetic and natural antioxidants. Fuel.

[69] García M., Botella L., Gil-Lalaguna N., Arauzo J., Gonzalo A., Sánchez J.L. (2017). Antioxidants for biodiesel: Additives prepared from extracted fractions of bio-oil. Fuel Process Technol..

[70] Gil-Lalaguna N., Bautista A., Gonzalo A., Sanchez J.L., Arauzo J. (2017). Obtaining biodiesel antioxidant additives by hydrothermal treatment of lignocellulosic bio-oil. Fuel Process Technol..

[71] Garcia-Perez M., Adams T.T., Goodrum J.W., Das K.C., Geller D.P. (2010). DSC studies to evaluate the impact of bio-oil on cold flow properties and oxidation stability of bio-diesel. Bioresour. Technol..

[72] Garcia-Perez M., Shen J., Wang X.S., Li C.Z. (2010). Production and fuel properties of fast pyrolysis oil/bio-diesel blends. Fuel Process Technol..

[73] Alcala A., Bridgwater A.V. (2013). Upgrading fast pyrolysis liquids: Blends of biodiesel and pyrolysis oil. Fuel.

[74] Xin J., Imahara H., Saka S. (2009). Kinetics on the oxidation of biodiesel stabilized with antioxidant. Fuel.

[75] Ashraful A., Masjuki H., Kalam M., Rahman S.A., Habibullah M., Syazwan M. (2014). Study of the effect of storage time on the oxidation and thermal stability of various biodiesels and their blends. Energy Fuel.

[76] Batista L., Da Silva V.F., Pissurno É.C., da Conceição Soares T., de Jesus M.R., Kunigami C.N., Brasil M.G., da Fonseca M.G. (2015). Formation of toxic hexanal, 2-heptenal and 2, 4-decadienal during biodiesel storage and oxidation. Environ. Chem. Lett..

[77] Christensen E., McCormick R.L. (2014). Long-term storage stability of biodiesel and biodiesel blends. Fuel Process Technol..

[78] Kulczynski B., Gramza-Michałowska A., Krolczyk J.B. (2020). Optimization of extraction conditions for the antioxidant potential of different pumpkin varieties (*Cucurbita maxima*). Sustain. Times.

[79] Rajendran S., Venkatesan E.P., Dhairiyasamy R., Jaganathan S., Muniyappan G., Hasan N. (2023). Enhancing performance and emission characteristics of biodiesel-operated compression ignition engines through low heat rejection mode and antioxidant additives: A Review. ACS Omega.

[80] Yang Z., Hollebone B.P., Wang Z., Yang C., Landriault M. (2013). Factors affecting oxidation stability of commercially available biodiesel products. Fuel Process Technol..

[81] Moser B.R. (2011). Influence of extended storage on fuel properties of methyl esters prepared from canola, palm, soybean and sunflower oils. Renew. Energy.

[82] Lau C.H., Gan S., Nang H.L.L., Lee L.Y. (2022). Insights into the effectiveness of synthetic and natural additives in improving biodiesel oxidation stability. Sustain. Energy Technol. Assess..

[83] Longanesi L., Pereira A.P., Johnston N., Chuck C.J. (2022). Oxidative stability of biodiesel: Recent insights. Biofuels Bioprod. Bioref..

[84] Sonam K.S., Guleria S. (2017). Synergistic antioxidant activity of natural products. Ann. Pharmacol. Pharm..

[85] Endo Y., Usuki R., Kaneda T. (1985). Antioxidant effect of chlorophyll and pheophytin on the autoxidation of oils in the dark. 1. Comparison of the inhibitory effects. J. Am. Oil Chem. Soc..

[86] Bart J.C.J., Palmeri N., Cavallaro S. (2010). Biodiesel Science and Technology from Soil to Oil.

[87] Komariah L.N., Arita S., Rendana M., Ramayanti C., Suriani N.L., Erisna D. (2022). Microbial contamination of diesel-biodiesel blends in storage tank; an analysis of colony morphology. Heliyon.

[88] Magalhaes A.M.S., Pereira E., Meirelles A.J.A., Sampaio K.A., Maximo G.J. (2019). Proposing blends for improving the cold flow properties of ethylic biodiesel. Fuel.

[89] Altaie M.A.H., Janius R.B., Rashid U., Taufiq Yap Y.H., Yunus R.R., Zakaria R. (2015). Cold flow and fuel properties of methyl oleate and palm-oil methyl ester blends. Fuel.

[90] Leggieri P.A., Senra M., Soh L. (2018). Cloud point and crystallization in fatty acid ethyl ester biodiesel mixtures with and without additives. Fuel.

[91] Moser B.R. (2009). Comparative oxidative stability of fatty acid alkyl esters by accelerated methods. J. Am. Oil Chem. Soc..

[92] Sundus F., Fazal M.A., Masjuki H.H. (2017). Tribology with biodiesel: A study on enhancing biodiesel stability and its fuel properties. Renew. Sustain. Energy Rev..

[93] Santos-Sanchez N.F., Salas-Coronado R. (2019). Antioxidant compounds and their antioxidant mechanism. IntechOpen.

[94] Ibrahim U.K., Kamarrudin N., Suzihaque M.U.H., Abd Hashib S. (2017). Local fruit wastes as a potential source of natural antioxidant: An overview. IOP Conf. Ser. Mater. Sci. Eng..

[95] Narayanasamy B., Jeyakumar N., Manoharan D.K. (2018). Effect of natural antioxidants on the oxidation stability of methyl ester of rubber seed oil. Energy Sources Part A Recover. Util. Environ. Eff..

[96] Pabon-Baquero L.C., Otalvaro-Alvarez A.M., Fernandez M.R.R., Chaparro-Gonzalez M.P. (2018). Plant extracts as antioxidant additives for food industry. Antioxid. Foods Appl..

[97] Ramos T.C.P.M., de Souza E.F., Pina C.C., Cavalheiro A.A., Fiorucci A.R., da Silva M.S. (2018). Evaluation of natural antioxidants action in oxidative stability of commercial biodiesel. Orbital Electron. J. Chem..

[98] Focke W.W., Van Der Westhuizen I., Grobler A.B.L., Nshoane K.T., Reddy J.K., Luyt A.S. (2012). The effect of synthetic antioxidants on the oxidative stability of biodiesel. Fuel.

[99] Jakeria M.R., Fazel M.A., Haseeb A.S.M.A. (2014). Influence of different factors on the stability of biodiesel: A review. Renew. Sustain. Energy Rev..

[100] Tang H., Wang A., Salley S.O., Simon-Ng K.Y. (2008). The effect of natural and synthetic antioxidants on the oxidative stability of biodiesel. J. Am. Oil Chem. Soc..

[101] Almeida E.S., Portela F.M., Sousa R.M., Daniel D., Terrones M.G., Richter E.M., Muñoz R.A. (2011). Behaviour of the antioxidant tert-butylhydroquinone on the storage stability and corrosive character of biodiesel. Fuel.

[102] Nagappan M., Devaraj A., Babu J.M., Saxena N.V., Prakash O., Kumar P., Sharma A. (2022). Impact of additives on combustion, performance and exhaust emission of biodiesel fueled direct injection diesel engine. Mater. Today.

[103] Ahmad Masudi A., Oki Muraza O., Che Jusoh N.W., Ubaidilla U. (2023). Improvements in the stability of biodiesel fuels: Recent progress and challenges. Environ. Sci. Pollut. Res..

[104] Embuscado M.E., Shahidi F. (2015). Herbs and spices as antioxidants for food preservation. Handbook of Antioxidants for Food Preservation.

[105] Fadda A. (2022). Innovative and sustainable technologies to enhance the oxidative stability of vegetable oils. Sustain. Times.

[106] Klaudia J., Michael L., Marian V., Bartosz G. (2014). Mechanisms of antioxidant activity. Food Oxidants and Antioxidants Chemical, Biological, and Functional Properties.

[107] Bassil D., Makrus D.P., Kefalas P. (2005). Oxidation of caffeic acid in the presence of cysteine: Isolation of 2, 5-cysteinylcaffeic acid and evaluation of its antioxidants properties. Food Res. Int..

[108] Damasceno S.S., Santos N.A., Santos I.M.G., Souza A.L., Souza A.G., Queiroz N. (2013). Caffeic and ferulic acids: An investigation of the effect of antioxidants on the stability of soybean biodiesel during storage. Fuel.

[109] Herbinet O., Pitz W.J., Westbrook C.K. (2010). Detailed chemical kinetic mechanism for the oxidation of biodiesel fuels blend surrogate. Combust. Flame.

[110] Moser B.R. (2008). Efficacy of myricetin as an antioxidant in methyl esters of soybean oil. Eur. J. Lipid Sci. Technol..

[111] Serqueira D.S., Dornellas R.M., Silva L.G., De Melo P.G., Castellan A., Ruggiero R., Richter E.M., Munoz R.A.A. (2015). Tetrahydro-curcuminoids as potential antioxidants for biodiesels. Fuel.

[112] Medeiros M.L., Cordeiro A.M.M.T., Queiroz N., Soledade L.E.B., Souza A.L., Souza A.G. (2014). Efficient antioxidant formulations for use in biodiesel. Energy Fuel.

[113] Spacino K.R., Silva E.T., Angilelli K.G., Moreira I., Galão O.F., Borsato D. (2016). Relative protection factor optimisation of natural antioxidants in biodiesel B100. Ind. Crops Prod..

[114] Deyab M.A. (2016). Corrosion inhibition of aluminum in biodiesel by ethanol extracts of Rosemary leaves. J. Taiwan Inst. Chem. Eng..

[115] Elavarasan P., Kondamudi K., Upadhyayula S. (2010). Synthesis of antioxidants: Green chemistry route. Int. J. Chem. Sci..

[116] Pullen J., Saeed K. (2012). An overview of biodiesel oxidation stability. Renew. Sustain. Energy Rev..

[117] Nakano M., Okawa K. (2014). Study of oxygen-containing hydrocarbons in exhaust emission from a spark ignition combustion engine. Int. J. Engine Res..

[118] Mwang J.K., Lee W.J., Chang Y.C., Chen C.Y., Wang L.C. (2015). An overview: Energy saving and pollution reduction by using green fuel blends in diesel engines. Appl. Energy.

[119] Song H., Quinton K.S., Peng Z., Zhao H., Ladommatos N. (2016). Effects of Oxygen Content of Fuels on Combustion and Emissions of Diesel Engines. Energies.

[120] Saluja R.K., Kumar V., Sham R. (2016). Stability of biodiesel–A review. Renew. Sustain. Energy Rev..

[121] Rizwanul Fattah I.M., Masjuki H., Kalam M., Hazrat M., Masum B., Imtenan S., Ashraful A. (2014). Effect of antioxidants on oxidation stability of biodiesel derived from vegetable and animal based feedstocks. Renew. Sustain. Energy Rev..

[122] Barclay L.R.C., Melinda R., Vinqvist M.R., Rappoport Z. (2003). Phenols as antioxidants. The Chemistry of Phenols.

[123] Li B., Pratt D.A. (2015). Methods for determining the efficacy of radical-trapping antioxidants. Free Radic. Biol. Med..

[124] Locomte J. (2010). Relationship between hydrophobicity and antioxidant ability of “phenolipids” in emulsion: A parabolic effect of the chain length of rosmarinate esters. J. Agric. Food Chem..

[125] Peltzer M.A., Wagner J.R., Migallon A.J., Zaigov G.E., Jiménez A. (2007). Stabilization of polymers with natural antioxidants. Polymer and Biopolymer Analysis and Characterization.

[126] Denisov E.T., Denisova T.G. (1999). Handbook of Antioxidants: Bond Dissociation Energies, Rate Constants.

[127] Hawkins W.L., Hawkins W.L. (1984). Stabilization against thermal oxidation. Polymer Degradation and Stabilization.

[128] Ingendoh A. (2010). Protection of biodiesel against oxidation. Lipid Technol..

[129] Chen Y.H., Luo Y.M. (2011). Oxidation stability of biodiesel derived from free fatty acids associated with kinetics of antioxidants. Fuel Process Technol..

[130] Gordon M., Hudson B.J.F. (1990). The mechanism of antioxidant action in vitro. Food Antioxidants.

[131] Zhong Y., Shahidi F. (2012). Antioxidant behavior in bulk oil: Limitations of polar paradox theory. J. Agric. Food Chem..

[132] Lapuerta M., Rodríguez-Fernández J., Ramos Á., Álvarez B. (2012). Effect of the test temperature and anti-oxidant addition on the oxidation stability of commercial biodiesel fuels. Fuel.

[133] Zuleta E.C., Rios L.A., Benjumea P.N. (2012). Oxidative stability and cold flow behavior of palm, sacha-inchi, jatropha and castor oil biodiesel blends. Fuel Process Technol..

[134] Hazrat M.A. (2020). A mini review on the cold flow properties of biodiesel and its blends. Front. Energy Res..

[135] Islam M.M., Hassan M.H., Kalam M.A., Binti N.W., Zulkifli M., Habibullah M., Hossain M.M. (2016). Improvement of cold flow properties of Cocos nucifera and Calophyllum inophyllum biodiesel blends using polymethyl acrylate additive. J. Clean. Prod..

[136] Boshui C., Yuqiu S., Jianhua F., Jiu W., Jiang W. (2010). Effect of cold flow improvers on flow properties of soybean biodiesel. Biomass Bioenergy.

[137] Muniz A.S. (2020). Bifunctional additives to improve the cold flow properties and oxidation stability of soybean oil biodiesel. Energy Fuel.

[138] Edith O. (2012). Factors affecting the cold flow behaviour of biodiesel and methods for improvement—A review, Pertanika. J. Sci. Technol..

[139] Anoop G.S., Hariprasad V., Deepak G.S., Akhil P.S. (2020). Enhancement of oxidative stability and cold flow properties of coconut oil using natural antioxidant additives for development of bio-lubricant. Proc. Inst. Mech. Eng. Part J J. Eng. Tribol..

[140] Rodrigues J.S. (2020). Comparative study of synthetic and natural antioxidants on the oxidative stability of biodiesel from Tilapia oil. Renew. Energy.

[141] Mittelbach M., Schober S. (2003). The influence of antioxidants on the oxidation stability of biodiesel. JAOCS J. Am. Oil Chem. Soc..

[142] Dunn R.O. (2005). Effect of antioxidants on the oxidative stability of methyl soyate (biodiesel). Fuel Process. Technol..

[143] Abril D. (2019). Comparison of the oxidative stability and antioxidant activity of extravirgin olive oil and oils extracted from seeds of *Colliguaya integerrima* and *Cynara cardunculus* under normal conditions and after thermal treatment. Antioxidants.

[144] Agarwal S., Singhal S., Singh M., Arora S., Tanwer M. (2018). Role of antioxidants in enhancing oxidation stability of biodiesels. ACS Sustain. Chem. Eng..

[145] Jahromi S.G. (2019). Extraction techniques of phenolic compounds from plants. Plant Physiol. Asp. Phenolic Compd..

[146] Neglo D. (2021). Comparative antioxidant and antimicrobial activities of the peels, rind, pulp and seeds of watermelon (*Citrullus lanatus*) fruit. Sci. Afr..

[147] Dai Y., Row K.H. (2019). Isolation and determination of beta-carotene in carrots by magnetic chitosan beta-cyclodextrin extraction and high-performance liquid chromatography (HPLC). Anal. Lett..

